# Wilson Disease: Uncommon but Not to Be Forgotten

**DOI:** 10.7759/cureus.39247

**Published:** 2023-05-19

**Authors:** Ahila Manivannan, Sanam Husain, Batool Shukr

**Affiliations:** 1 Internal Medicine, Henry Ford Health System, Detroit, USA; 2 Pathology, Henry Ford Health System, Detroit, USA

**Keywords:** liver transplant, peth, alcohol use disorder (aud), cirrhosis, wilson disease

## Abstract

Wilson disease (WD) is an autosomal recessive genetic disorder caused by mutations of ATP7B, a copper transporter, which results in impaired copper clearance. Its clinical manifestations are varied and can result in a mix of hepatic and neuropsychiatric symptoms. We present the case of a 26-year-old female with a past medical history of alcohol use who presented with right upper quadrant abdominal pain with associated vomiting, jaundice, and fatigue. She was found to have signs and symptoms of decompensated cirrhosis and was initially concerned about superimposed alcoholic hepatitis. With low ceruloplasmin and alkaline phosphatase, the suspicion for WD remained, and the patient underwent liver transplantation due to her worsening clinical status. The quantitative hepatic copper content of the explanted liver was elevated, and genetic testing confirmed the diagnosis of WD. Our case highlights the importance of including WD in the differential of a young patient with severe liver disease, and it highlights the utility of the phosphatidyl ethanol (PEth) test as a marker for chronic severe alcohol use. In patients with a significant alcohol use history, the diagnosis of WD should still be considered for those with reasonable clinical suspicion.

## Introduction

Wilson disease (WD) is an autosomal recessive genetic disorder caused by mutations of the ATP7B gene, which encodes the intracellular copper transporter ATP7B. Mutations of this gene lead to impaired copper clearance. Copper accumulates in tissues, mainly the liver and central nervous system, which leads to cirrhosis and neuropsychiatric effects. WD is a relatively rare disease, with a prevalence of about 1 per every 30,000 [1]. There is a broad spectrum of hepatic manifestations of this disease, ranging from asymptomatic hepatomegaly to persistently elevated liver function tests (LFTs) to cirrhosis and acute liver failure. Some of these patients can present with symptoms that closely resemble acute viral hepatitis or autoimmune hepatitis as well [2].

Tests that aid in the diagnosis of WD include aspartate aminotransferase (AST) levels higher than alanine aminotransferase (ALT) levels, especially in cirrhosis; low alkaline phosphatase (ALP); low serum ceruloplasmin (less than 20 mg/dl); and Kayser Fleischer rings noted on a slit lamp. Low ceruloplasmin itself is not enough to make a diagnosis of WD, as a liver biopsy with quantification of liver copper levels, basal 24-hour urinary copper excretion, and genetic testing are usually recommended [2]. Scoring systems such as the Revised Wilson’s Index and the Leipzig score can be used to aid in the diagnosis of WD as well, which consider specific clinical features and laboratory tests [3]. Without lifelong oral therapies or a liver transplant in severe cases, WD is fatal if left untreated. There is often a delay in the diagnosis of this disease due to its often confounding presentation, which emphasizes the importance of keeping WD in the differential.

## Case presentation

We present the case of a 26-year-old Caucasian female who presented with a two-week history of progressively worsening right upper quadrant abdominal pain with associated vomiting, jaundice, and fatigue. She had no family history of liver disease. She endorsed a history of alcohol use starting at the age of 18, with an average of two to four drinks per week, including binge drinking episodes, with the last episode occurring three weeks prior to the presentation. She did not have any other obvious risk factors for liver disease, such as over-the-counter medications or supplement use. The physical exam was notable for jaundice and right upper quadrant abdominal pain. Initial laboratory work is demonstrated in Table 1.

**Table 1:** Laboratory values on admission and after one week

	Initial laboratory values	After one week into admission
Sodium (mmol/L)	134	127
Creatinine (mg/dL)	0.76	2.38
Hemoglobin (g/dL)	11.3	7.2
Platelet count (k/µL)	65	26
Alanine aminotransferase (IU/L)	45	29
Aspartate aminotransferase (IU/L)	116	112
Total bilirubin (mg/dL)	7.6	67.2
Alkaline phosphatase (IU/L)	45	14
International normalized ratio	2.9	6.11

**Figure 1:**
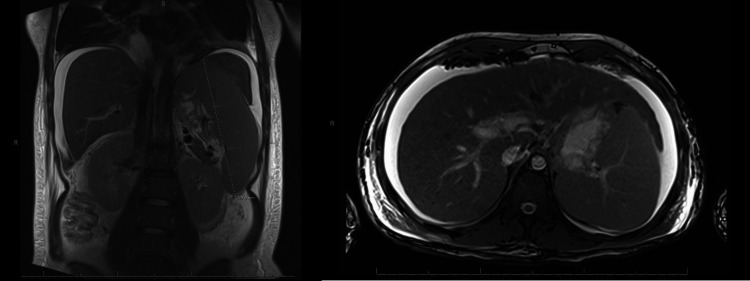
Coronal and axial images from MRCP demonstrating subtle liver surface nodularity and linear fibrosis with signs of portal hypertension, including moderate ascites and splenomegaly up to 18.8 cm.

The viral hepatitis screen was negative, and in autoimmune liver studies, acetaminophen and salicylate levels were normal. Ceruloplasmin was low at 10 mg/dL (reference range 20-60). As seen in Figure 1, magnetic resonance cholangiopancreatography (MRCP) demonstrated subtle liver surface nodularity and linear fibrosis, consistent with cirrhosis, portal hypertension, and ascites.

Due to the severity of her initial presentation, she was transferred to a liver transplant center within two days of her presentation. One week into admission, her clinical status continued to decline with worsening liver and renal function. Repeat laboratory studies are shown in Table 1, corresponding to a MELD-Na score greater than 40. There was also suspicion of hemolysis given her haptoglobin level was less than 30 mg/dL (reference range 30-200 mL/dL) and her LDH was 387 IU/dL (reference range <250 IU/dL). The underlying etiology of her cirrhosis was unclear, and while acute or chronic alcoholic liver disease was suspected to be the cause, her labs were suggestive of fulminant hepatic failure possibly secondary to WD given the low ceruloplasmin and ALP. The slit lamp examination was negative for Kaiser Fleischer rings. Due to the severity of her condition and her coagulopathy, which prevented a safe liver biopsy, she underwent an expedited liver transplant workup. The patient underwent liver transplantation 12 days after her initial presentation, prior to having a confirmed etiology for her liver failure. Phosphatidylethanol (PEth) testing, which can detect excessive alcohol intake within a two-week period, resulted in a negative result after her transplant, indicating alcohol was unlikely to be the etiology of her liver disease.

**Figure 2:**
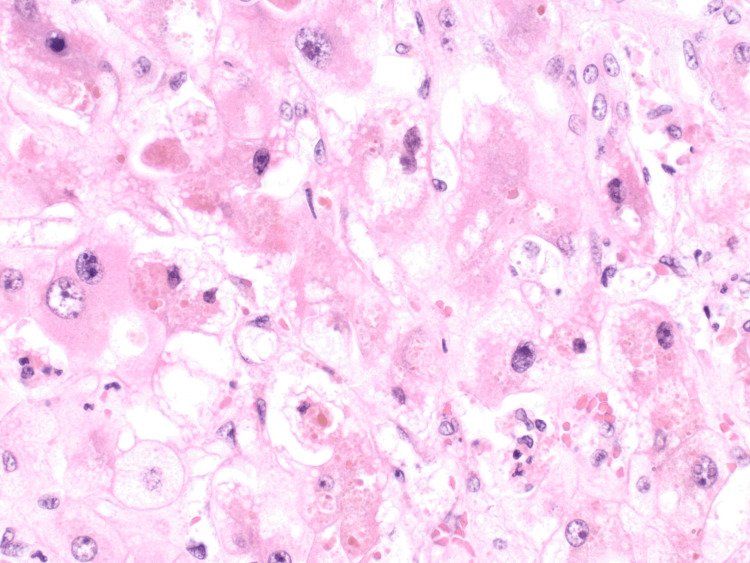
Histology slide of the patient’s explanted liver, demonstrating steatohepatitis and Mallory-Denk bodies.

The histologic image in Figure 2 from the patient’s explanted liver demonstrated evidence of steatohepatitis and Mallory-Denk bodies, features that can be seen in alcoholic and non-alcoholic fatty liver disease.

The hepatic copper content of the explanted liver was 1544 H mcg/g dry weight (ref range: 10-35), strongly suggestive of WD. Genetic testing demonstrated the patient was heterozygous for both a pathologic variant (H1069Q) and a likely pathologic variant (G691V) of the ATP7B gene, consistent with the diagnosis of WD. The patient is doing well after the transplant and has had no significant complications.

## Discussion

This case highlights the importance of including WD in the differential diagnosis of fulminant liver failure and decompensated cirrhosis. For patients with WD presenting with severely decompensated liver disease or liver failure, liver transplantation is lifesaving and curative. These patients generally do very well after transplant and have been noted to have improved survival after transplant compared to other patients with acute liver failure. Patients suspected to have WD in these cases should be transferred to a liver transplant quickly, as this patient was [4].

It is important to note that ceruloplasmin is limited as a diagnostic tool. In our patient, WD was on the differential due to low ceruloplasmin; however, her alcohol history and lack of Kayser Fleischer rings were misleading. It is interesting to note that while our patient did not have Kayser Fleischer rings, that is not an uncommon finding as only 65% of patients with hepatic manifestations will present with these rings, compared to 95% of patients with WD who present with neuropsychiatric manifestations [5]. Her low ceruloplasmin had initially been attributed to her malnourished condition. Ceruloplasmin can be falsely low in a variety of conditions, such as protein-losing enteropathy and severe chronic liver disease with reduced synthetic function, among others [2]. One case report describes a patient initially thought to have alcoholic cirrhosis whose ceruloplasmin level was initially normal, but when readmitted for worsening liver disease, he was found to have low ceruloplasmin and was ultimately diagnosed with WD [6]. In 20% of patients with WD, ceruloplasmin may actually be at the lower limit of normal, which may deter clinicians from making that diagnosis [7].

A highlight of this case is the fact that our patient's clinical presentation was confounded by her alcohol use history. A PEth test, if ordered sooner, could have helped further point towards Wilson's disease. Phosphatidylethanol, which is a byproduct formed on the phospholipid membrane of red blood cells in the presence of alcohol, is a good biomarker for identifying recent chronic alcohol use. PEth levels reflect alcohol use as far back as three weeks and are not affected by organ dysfunction or demographics like race or age. With high sensitivity and specificity, it is a better test than other alcohol biomarkers for assessing heavy chronic alcohol use, as seen in our case [8]. It is possible that our patient had some component of underlying alcoholic liver disease as well, exacerbated by this acute presentation. There have been details of other cases of patients with significant alcohol use eventually being diagnosed with WD, leading to the suspicion that there may be cases of WD with superimposed alcohol-related liver disease [9]. Our case also underscores the importance of a detailed alcohol use history and the need to suspect WD in younger patients even when other etiologies are suspected.

## Conclusions

Our case highlights the importance of including WD in the differential of a young patient with severe liver disease, and it highlights the utility of the PEth test as a marker for chronic severe alcohol use. As our patient was transferred to a liver transplant center early on in her course, she was able to undergo a timely life-saving liver transplant, which emphasizes the importance of promptly transferring these patients to a liver transplant center. In patients with a significant alcohol use history, the diagnosis of WD should still be considered in those with reasonable clinical suspicion. Retrospective studies should be conducted to analyze the coexistence of alcohol use disorder and WD and how that may affect the patient’s clinical trajectory.
